# Development and validation of a multiplex-PCR assay for X-linked intellectual disability

**DOI:** 10.1186/1471-2350-14-80

**Published:** 2013-08-05

**Authors:** Paula Jorge, Bárbara Oliveira, Isabel Marques, Rosário Santos

**Affiliations:** 1 Centro de Genética Médica Dr. Jacinto Magalhães, CHP, Praça Pedro Nunes 88, 4099-028, Porto, Portugal; 2 New address: Instituto Nacional de Saúde Dr. Ricardo Jorge INSA I.P., Lisbon, Portugal

**Keywords:** X-linked intellectual disability (XLID), *FMR1*, *AFF2*, *ARX*, Multiplex-PCR

## Abstract

**Background:**

X-linked intellectual disability is a common cause of inherited cognitive deficit affecting mostly males. There are several genetic causes implicated in this condition, which has hampered the establishment of an accurate diagnosis. We developed a multiplex-PCR assay for the mutational hotspot regions of the *FMR1*, *AFF2* and *ARX* genes.

**Methods:**

The multiplex-PCR was validated in a cohort of 100 males selected to include known alleles for the *FMR1* repetitive region: five full mutations (250–650 CGGs), ten premutations (70–165 CGGs) and eighty-five in the normal range (19–42 CGGs). Sequencing or Southern blotting was used to confirm the results, depending on the allele class. In this cohort, with the exception of one sample showing an *AFF2* intermediate-sized allele, all other samples were normal (8–34 CCGs). No *ARX* variant was found besides the c.429_452dup. The validated assay was applied to 5000 samples (64.4% males and 35.6% females).

**Results:**

The normal-allelic range of both *FMR1* and *AFF2* genes as well as the nature of *ARX* variants identified was similar in both genders. The rate of homozygosity observed in female samples, 27.5% for *FMR1* and 17.8% for *AFF2* alleles, is comparable to that published by others. Two *FMR1* premutations were identified, in a male (58 CGGs) and a female case [(CGG)_47_/(CGG)_61_], as well as several *FMR1* or *AFF2* intermediate-sized alleles. One *AFF2* premutation (68 CCGs) and two putative full expansions were picked up in male subjects, which seems relevant considering the rarity of reported *AFF2* mutations found in the absence of a family history.

**Conclusions:**

We developed a robust multiplex-PCR that can be used to screen the mutational hotspot regions of *FMR1*, *AFF2* and *ARX* genes. Moreover, this strategy led to the identification of variants in all three genes, representing not only an improvement in allele-sizing but also in achieving a differential diagnosis. Although the distinction between females who are truly homozygous and those with a second pre- or full mutation sized allele, as well as a definitive diagnosis, requires a specific downstream technique, the use of this multiplex-PCR for initial screening is a cost-effective approach which widens the scope of detection.

## Background

X-linked intellectual disability (XLID) is a group of genetically heterogeneous disorders caused by mutations in X-chromosome genes. Currently, it is believed that X-linked recessive gene defects account for about 10–12% of overall intellectual disability (ID) seen in males [1]. The lack of a comprehensible relationship between clinical signs and genotype in XLID, together with the large number of genes implicated, are the major obstacles in their genetic diagnosis and places ID as one of the most important unsolved problems in healthcare. Nowadays, a huge amount of information can be generated by assessing ID from both the clinical and molecular point of view. However, the challenge remains as to how all of this information can be used to establish a simple, standard and effective strategy applicable to molecular diagnosis and genetic counselling. Among the genetic causes involved in XLID, mutations in the genes *FMR1* (fragile X mental retardation 1) and *ARX* (aristaless related homeobox) emerge as important causes. *AFF2* (AF4/FMR2 family, member 2) testing is often requested when patients test negative for *FMR1*, particularly in the absence of specific syndromic features [2-4].

The *FMR1* gene contains polymorphic repetitive regions susceptible to suffer dynamic mutations, a process that may give rise to pathogenic expansions. The expansion to over 200 CGG repeats in the *FMR1* gene is associated with fragile X syndrome [FXS, FRAXA; MIM#300624], the most common form of familial severe XLID [5,6]. Below 200 repeats, alleles are classified as normal (5–44 CGGs), intermediate (45–54 CGGs) or premutated (55–200 CGGs) (see also the *Technical Standard and Guideline for Fragile X Testing*, http://www.acmg.net/Pages/ACMG_Activities/stds-2002/fx.htm, 2006 edition, last accessed September 2012) [7].

Besides the X-chromosome fragile site A, fragile site E (locus in Xq28) is also associated with intellectual disability [FRAXE; MIM #309548]. This disorder is mainly a non-syndromic form of X-linked intellectual disability and is the result of silencing of the *AFF2* gene, as a consequence of an upstream CCG expansion [8]. In the normal population, the number of CCG repeats varies between 6 and 35, while it is increased to more than 200 hyper-methylated triplets in FRAXE/*AFF2* intellectually disabled patients. In contrast to FXS, reports of *AFF2* full expansions are very rare and the dynamics of this repeat is not as clearly characterized as that of *FMR1*. Indeed, to what extent the alleles with CCG repeats in the range between 36 and 199 may exhibit a pathogenic role remains elusive, in part due to the rarity of the mutation and extended pedigrees [9].

In the *ARX* gene, the second exon represents a mutational hotspot as it contains repetitive regions that codify alanines. Since its identification, several mutations have been identified, including insertions, deletions, duplications, missense, nonsense and splice mutations, leading to a wide spectrum of phenotypes that include syndromic as well as nonsyndromic forms of intellectual disability [10]. These different mutations were reported in patients with over 10 distinct clinical outcomes. Even though the mutations described extend across the gene, the majority occur in the largest coding exon of the *ARX* gene, exon 2, where variations in polyalanine tracts, are among the most frequent [11,12]. Indeed the c.429_452dup mutation, affecting the second tract, is the most frequent *ARX* mutation [13]. The absence of genotype/phenotype correlation in the *ARX* gene explains our inability to establish the respective guidelines for molecular screening particularly in XLID patients that do not include brain malformations.

As the early recognition of syndromic physical traits is difficult, it is widely accepted that boys with unexplained ID/developmental delay, with or without any other clinical findings, should be screened for FXS. The literature supports the benefit of a screening method due to its prevalence and the fact that molecular genetic testing for FXS is highly sensitive and specific [14]. To the best of our knowledge there is no reference to a three-gene approach to the diagnosis of ID, specifically one that covers two of the most frequently mutated XLID genes. To address this issue we have developed a molecular test based on a multiplex-PCR, focusing on mutational hotspots of *FMR1*, *AFF2* and *ARX* genes and validated in a male cohort. Using this new assay we screened 5000 samples from male and female subjects referred for FXS testing and have widened the identification of the genetic causes of some XLID cases.

## Methods

All genomic DNA (gDNA) samples had previously been extracted by the salting out method, from peripheral blood collected in ethylenediamine tetra acetic acid (EDTA), dissolved in Tris-EDTA buffer (pH = 7) and stored at 4°C [15]. No patients were recruited specifically for this study; all samples belong to subjects previously referred by medical geneticists for fragile X syndrome (FXS) molecular testing [16]. Their phenotypic spectrum ranged from severe intellectual disability to language delay or learning disabilities with normal to borderline psychomotor development. Approval for this study was obtained from Ethics committee of the Instituto Nacional de Saúde Dr. Ricardo Jorge (INSA, I.P.). The participants, or their legally authorized representatives, had given informed consent to take part in this research.

A validation cohort consisting of 100 male gDNAs was chosen to include normal *FMR1* alleles, ten *FMR1* premutations and five full mutations. A sample with the *ARX* mutation, c.429_452dup, was also used. This mutation had previously been published and described as c.428_451dup24 [17]. The test group comprised 5000 gDNAs of which 64.4% (n = 3220) were males and 35.6% (n = 1780) females.

The method developed consisted in the combination of three primer pairs that amplify target regions, as follows: *FMR1*, forward 6-FAM™ labelled 5′ CCA TCT TCT CTT CAG CCC TGC 3′ and reverse 5′ TTC GGT TTC ACT TCC GGT G 3′; *AFF2*, forward HEX labelled 5′ TGT GAG TGT GTA AGT GTG TGA TGC TGC C 3′ and reverse 5′ CCG CGC GCA CCC AGC GAC 3′ and *ARX*, forward 5′ CAG CAG CCC TGG CTG GGA CTC 3′ and reverse HEX labelled 5′ CGG TAC GAC TTG CTG CGG CTG 3′. While *FMR1* and *AFF2* amplicons encompass the CGG and CCG polymorphic triplet repeats, respectively, the *ARX* exon 2 PCR (*ARX* ex2p), is designed to amplify a portion of the second exon of *ARX* gene, that includes two of the three stretches of repetitive alanine coding triplets (poly AI and AII); a 380 bp amplicon corresponding to the normal allele is produced or, in the presence of the c.429_452dup, a 404 bp amplicon. First, the optimization process was done using each pair of primers in a separate amplification reaction and tested for annealing temperatures ranging from 55°C to 62°C. PCR components were then tested in a multiplexed reaction, one by one, in an empirically decided order, namely primer amounts, extension time and concentration of several PCR co-adjuvants such as 7-deaza dGTP, betaine and DMSO. The optimized multiplex-PCR mixture prepared in a final volume of 25 μL contained a final concentration of 1 M betaine, 0.12 mM each dATP, dCTP and dTTP (Bioline, London, UK), 0.02 mM dGTP (Bioline, London, UK), 0.4 mM 7-deaza dGTP, Li-Salt (Roche, Indianapolis, USA), 4.8% DMSO (Sigma-Aldrich Co, St. Louis, USA), 10 pmol *FMR1* primers, 6 pmol *AFF2* primers, 5 pmol *ARX* primers and 1× Accutaq buffer (Sigma-Aldrich Co, St. Louis, USA). This mixture was supplemented with 1U AccuTaq polymerase (Sigma-Aldrich Co, St.Louis, USA) and a total of 300 ng of gDNA was added at the end. The amplification reaction was performed in a 9800 Fast Thermal Cycler (Applied Biosystems, Foster City, CA, USA). After an initial denaturation step for 10 minutes at 98°C, 42 cycles of amplification were performed as follows: denaturation at 98°C for 45 seconds, annealing at 58°C for 45 seconds and extension at 68°C for 2 minutes and 30 seconds, followed by an additional cycle of 10 minutes at 68°C for the final extension. PCR products were diluted at 1:15 in a reaction solution containing 0.5 μL GeneScan™ 500 ROX or MapMarker-1000-ROX, MapMarker®Size Std (BioVentures, Inc., Murfreesboro, TN, USA) and 13.5 μL Hi-Di formamide (Applied Biosystems, Foster City, CA, USA). Fragments were resolved on the 3130×l Genetic Analyser and further analysed using the GeneMapper® Software v4.0 (Applied Biosystems, Foster City, CA, USA).

Two types of methodologies were used to confirm the results obtained in the validation cohort, according to the allele class: sanger dideoxy sequencing for the amplifiable fragments, using primer sets upstream and downstream the *FMR1* CGG, or *AFF2* CCG repetitive regions, or the *ARX* ex2p, and Southern blotting, for the non-amplifiable fragments or alleles that fall into the pathogenic range. Sequencing was performed using BigDye® Terminator v1.1 Cycle Sequencing Kit (Applied Biosystems, Foster City, CA, USA) following the manufacturer’s indications with the primers described above (without the fluorescent labelling) and standard amplification conditions. Sequencing reactions were resolved on a 3130×l Genetic Analyser. The resulting data were further analysed using the SeqScape® Software v2.5 (Applied Biosystems, Foster City, CA, USA). Southern blot genotyping was done using ~10 μg of gDNA double-digested with the restriction enzymes EcoRI and EagI for *FMR1* gene or AflIII and NotI for *AFF2* gene (New England Biolabs, Ipswich, MA, USA). Further, the digested gDNA products were electrophoresed in parallel with a 1:1 mixture of the standard size markers, DNA Molecular Weight Marker II and III digoxigenin-labeled (Roche Applied Science, Indianapolis, IN, USA), blotted and hybridized using the *FMR1* or the *AFF2* digoxigenin-labeled specific probes, GLFXDIG1 or AJ31Dig1, as appropriate (Gene Link™, Hawthorne, NY, USA).

## Results and discussion

A screening strategy is described that allows the characterization of *FMR1* and *AFF2* repetitive regions in normal to premutation ranges together with the detection of size variants in the second exon of *ARX* gene. The amplification of *FMR1* CGG repetitive region has been widely tested for several PCR conditions, either changing polymerase or adding co-adjuvants [18-21]. Due to the high GC content in all three amplified fragments and particularly in the repetitive amplified regions (of *FMR1* and *AFF2* genes) leading to stutter band formation, a directly comparative quantitative result was not consistently obtained. Instead, we observed reduction in stutter band formation particularly in *FMR1* alleles, in amplifications using this multiplex-PCR, when compared to simplex reactions. Moreover, the simultaneous amplification of two similar fragments (*FMR1* and *AFF2* polymorphic regions), in terms of GC content and presence of triplet repeats, provides an internal amplification control.

**Figure 1:**
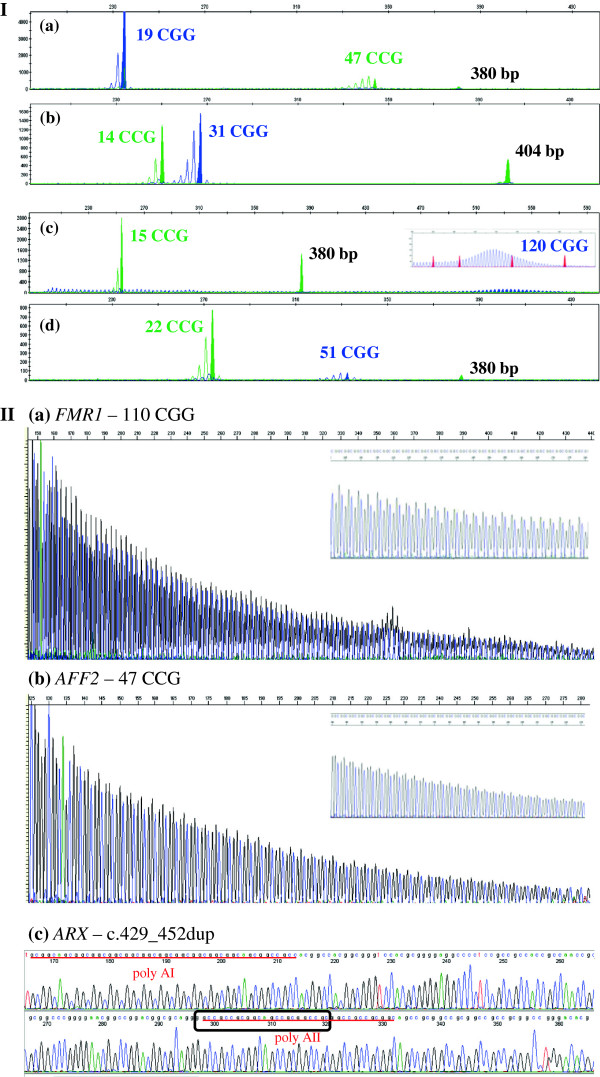
**Examples of validation cohort results. I-**Multiplex-PCR assay for the analysis of the mutational hotspot of *FMR1* CGG alleles (blue label), *AFF2* CCG alleles (green label) and *ARX* ex2p (380 bp green fragment). Profile: **(a)***AFF2* intermediate allele (47 CCGs); **(b)** c.429_452dup of *ARX* gene; **(c)***FMR1* premutation (120 CGGs); **(d)** FXS male mosaic with an *FMR1* intermediate allele (51 CGGs) and a full mutation. **II-**Sequencing results: **(a)** CGG repetitive region of *FMR1* gene (sense strand) showing the reproducibly sequenced premutated allele with 110 triplets; **(b)***AFF2* intermediate allele (47 CCGs) (reverse strand); **(c)** Partial nucleotide sequence of the *ARX* gene exon 2 (sense strand), showing the prevalent *ARX* mutation c.429_452dup. The box indicates the most upstream twenty-four base pair sequence that is duplicated. The two stretches of repetitive triplets coding for alanine (poly AI and AII) are underlined.

**Figure 2:**
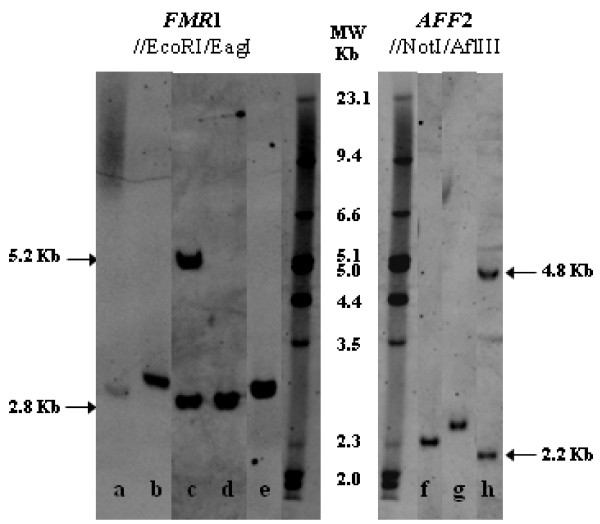
**Examples of Southern blot results.** Genotyping using *FMR1* probe: After EcoRI /EagI double digestion, normal females exhibit two fragments, a ~2.8 kb (active X) and a ~5.2 kb (inactive X), while in normal males only a ~2.8 kb fragment is seen: **(a)** FXS male mosaic for a 51 CGG allele and a full mutation; **(b)** Premutated *FMR1* male (110 CGGs); **(c)** Klinefelter with two *FMR1* normal sized-alleles, showing the active and the inactive X-chromosome; **(d)** Normal *FMR1* male (19 CGGs); **(e)** Premutated *FMR1* male (58 CGGs). Genotyping using *AFF2* probe: Following AflIII /NotI double digestion, normal females present two fragments, a ~2.2 kb (active X) and a ~4.8 kb (inactive X), whereas normal males exhibit only a ~2.2 kb fragment: **(f)** Intermediate *AFF2* male (47 CCGs); **(g)** Premutated *AFF2* male (68 CCGs); **(h)** Homoallelic female [(CCG)_14_/(CCG)_14_].

The validation cohort consisting of 100 male gDNAs was sequenced across the *FMR1* and *AFF2* repetitive regions and the *ARX* ex2p, used for allele number standardization for each gene and for further validation of the multiplex-PCR methodology. Table 1 compiles these results. Examples of the optimized multiplex-PCR, sequencing and Southern blot results are shown in Figures 1 and 2. Overall, *FMR1* CGG repeat sizes ranged from 19 to over 120, where the 30 CGG repeat allele represented 37% of the observed alleles. *AFF2* CCG repeat sizes ranged from 8 to 47 and alleles with 14 triplets represented 36% of the pool [Figure 1-I(a) and II(b), Figure 2(f)]. Besides, no other *ARX* sequence variant was observed [Figure 1-I(b) and II(c)].

**Table 1:** Validation cohort results

**n**	***FMR1*CGG**	***AFF2*CCG**	***ARX*(bp)**	**n**	***FMR1*CGG**	***AFF2*CCG**	***ARX*(bp)**	**n**	***FMR1*(CGG)**	***AFF2*(CCG)**	***ARX*(bp)**	**Obtained/confirmed by Southern bloting**
1	19	13	380	3	30	11	380	1	33	22	380	
1^a^	19	47	380	4	30	13	380	1	35	34	380	
2	20	14	380	1^b^	30	14	404	2	36	14	380	
1	20	15	380	14	30	14	380	1	37	23	380	
1	21	14	380	1	30	15	380	1	41	23	380	
1	21	15	380	1	30	16	380	1	42	23	380	
1	21	23	380	4	30	17	380	1	**65**	14	380	~70
3	23	14	380	2	30	18	380	1	**71**	14	380	~70
1	23	16	380	1	30	19	380	1	**75**	23	380	~80
2	23	17	380	2	30	20	380	1	**80**	22	380	~80
2	23	19	380	1	30	22	380	1	**85**	14	380	~80
1	23	20	380	1	30	24	380	1	**100**	15	380	~100
1	24	14	380	2	30	27	380	1	**110**	14	380	~100
1	27	14	380	1	31	14	380	1	120	15	380	~120
1	28	20	380	2	31	17	380	1	- (150)	21	380	~150
1	29	8	380	1	31	20	380	1	-	17	380	~165
3	29	14	380	1	31	21	380	1	-	16	380	~250
1	29	15	380	1	31	30	380	1^c^	51	22	380	~350
1	29	16	380	2	32	14	380	1	-	17	380	~350
1	29	9	380	1	32	15	380	1	-	14	380	~400
4	29	17	380	1	32	19	380	1	-	13	380	~650

Number of triplets obtained for *FMR1* and *AFF2* genes, and *ARX* ex2p fragment size, in 100 male samples. The order is according to *FMR1* allele sizes. Number of triplets obtained for *FMR1* and *AFF2* genes, and *ARX* ex2p fragment size, in 100 male samples, ordered according to *FMR1* allele sizes. [n] Number of samples per allele profile; ^a^*AFF2* intermediate size allele; ^b^control for the *ARX* c.429_452dup; ^c^FXS sample with mosaicism for an intermediate-size allele and a full mutation; Premutations clearly amplified by the multiplex-PCR and additionally confirmed by sequencing are bold highlighted; [−] not (reproducibly) amplified.

Samples with premutations and full mutations were on purpose included in the validation process to test for *FMR1* primer specificity in the multiplex-PCR assay [Figure 1-I(c)]. Expanded alleles were not the main purpose of this assay and presented inconsistent amplification results. Thus, the 110 CGG allele was settled as the reliable upper-boundary for the sequencing validation [Figure 1-II(a), Figure 2(b)]. Furthermore, whenever the multiplex-PCR assay revealed either absence or an expanded allele, the corresponding sample was subjected to Southern blot analysis. An obvious gain when using the multiplex-PCR, comparatively to Southern blotting, is the accuracy with which the allele-sizes are quantified, with emphasis in the clinically significant borderline alleles (Table 1).

In a sample belonging to a FXS patient (banked as a full mutation carrier) the multiplex-PCR assay revealed a fragment corresponding to a 51 CGG allele [Figure 1-I(d)]. Having excluded a sample identification error, another possible explanation for that finding, is that the blot (performed more than 16 years ago with an *FMR1* specific ^32^P-labelled probe) may not have had sufficient sensitivity to pick up low levels of mosaicism or the expected intermediate band could have become imperceptible to the naked eye. With the aim of validating this result, an additional Southern blot analysis was performed in which, besides the full mutation, a faint intermediate-sized band, was observed, consolidating the multiplex-PCR result [Figure 2(a)]. This example illustrates that this assay or any other PCR-based methodology used as a screening test *per se*, is unable to exclude size-mosaicism. As such, it is recommended to exclude the presence of size-mosaics resorting to a different methodology(e.g. Southern blot), particularly in the presence of strong clinical features or FXS family history.

**Figure 3:**
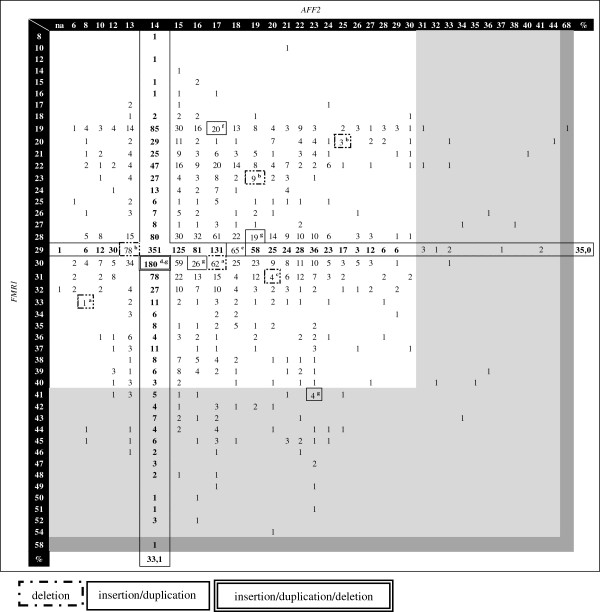
**Test group results.** Number of male samples *per* allele profile of both *FMR1* and *AFF2 loci*. Intermediate alleles are grey shaded. In dark grey the premutations identified in both *FMR1* and *AFF2* are shown. Percentage of the most frequently identified allele for both genes is indicated. Cases with different *ARX* variants are surrounded by boxes. [na] – absence of amplification for *AFF2* gene. *ARX* variant type: ^a^9 bp deletion (two samples); ^b^15 bp deletion (three samples); ^c^24 bp deletion; ^d^33 bp deletion; ^e^3 bp insertion/duplication; ^f^12 bp insertion/duplication; ^g^24 bp insertion/duplication (four subjects). Klinefelter cases are not represented as the allelic phase was unknown.

The validated multiplex-PCR assay was applied to 5000 samples (test group), previously screened for *FMR1* expansions by Southern blot methods only. 64.4% of the analysed alleles were from male samples (n = 3220) (Figure 3). In this subset, the *FMR1* alleles ranged from 8 to 58 repeats, where the 54 CGG allele is the largest within the normal range, while *AFF2* alleles ranged from 6 to 68 CCGs, being the 44 repeat allele the largest among the normal. Alleles containing 29 CGG repeats and 14 CCG repeats were seen to be the most frequent, representing 35.0% and 33.1% of the *FMR1* and *AFF2* alleles respectively. *FMR1* male intermediate-sized alleles (41 to 54 CGGs) revealed a frequency of 3.2%, while for *AFF2* gene only 0.71% fall into this category (31 to 44 CCGs). Although a direct comparison is difficult, these frequencies are comparable to those described in a similar survey focusing on the parameters used for the sample selection (using an inclusive rather than an exclusive selection) [9]. In a more recent study of a male subject control population, higher frequencies were obtained: 6% for the *FMR1* and 2% for the *AFF2* intermediate-sized alleles (Supplemental Table one in [22]). However, in that report a comparatively small number of samples were analysed (n = 124).

**Figure 4:**
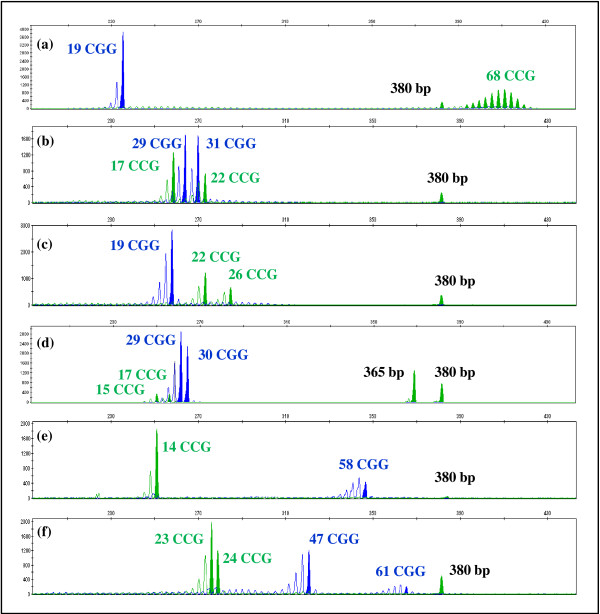
**Examples of test group results.** Multiplex-PCR assay focusing the analysis of the mutational hotspot of *FMR1* CGG alleles (blue label), *AFF2* CCG alleles (green label) and of the *ARX* ex2p (380 bp green fragment). Profiles: **(a)***AFF2* premutation male (68 CCGs); **(b)** and **(c)** male samples where the karyotype revealed the presence of an extra X-chromosome (47, XXY). Additionally in **(c)** an *FMR1* full mutation was subsequently identified by Southern blotting. Profiles: **(d)** Heterozygous *ARX* exon 2 deletion female; **(e)** and **(f)**: *FMR1* premutation alleles, male (58 CGGs) and female [(CGG)_47_/(CGG)_61_]. Both *FMR1***(d)** and *AFF2***(f)** alleles differing in size by a single triplet can be easily distinguished.

Regarding the *AFF2* gene, this multiplex-PCR assay revealed two presumed full mutations (2 in 3220), in which there was an unsuccessful amplification of the *AFF2* allele and a case with an allele in the premutation range with 68 CCG repeats (1 in 3220) [Figure 2(g), Figure 3 and Figure 4(a)]. A definitive diagnosis of these cases requires a specific downstream technique; hence analyses by Southern blot using an *AFF2* specific probe, as well as familial studies are being performed. The putative association of *AFF2* premutated alleles with the development of Parkinson disease in adulthood, the risk of expansion to pathogenic mutations in future generations as well as non-syndromic/non-pathognomonic clinical signs in *AFF2* mutation carriers, justifies mutation screening for this gene and are obvious benefits of *AFF2* gene analysis included in this multiplex-PCR [22].

Co-amplification of *AFF2* and *FMR1* has also proven useful in detecting X-chromosome aneuploidy. In informative samples when the number of amplified fragments is inconsistent with the number of expected X-chromosomes (according to patient gender) or there appears to be a discrepancy between the number of alleles for the different genes, further investigation must be done. In fact, we found nineteen of such cases in which the karyotype subsequently confirmed a Klinefelter syndrome, representing 0.59% of the analysed male samples, a frequency similar to others previously reported [Figure 2(c), Figure 4(b) and (c)] [9]. Surprisingly, in a 47, XXY case, a fully-methylated *FMR1* gene mutation was recently identified (data not shown).

In the male subset, thirteen size-variants were observed in the *ARX* gene by fragment analysis, including seven deletions (ranging from 9 to 33 bp) and six insertions or duplications (represented by an increase in size of 3 to 24 bp) (Figure 3). Subsequent sequencing revealed four cases with the known *ARX* mutation (c.429_452dup) as well as several other variants that change the alanine content of the ARX protein. Segregation studies and genotype/phenotype correlations are being completed. *ARX* pathogenic variants characterized to date are the cause of at least ten clinically distinct conditions, where intellectual disability is the cardinal feature [10]. The vast majority of mutations occurring outside known ARX domains or in domains other than the polyalanine (poly A) tracks are known to cause the most severe forms of *ARX* related disorders (e.g. X-linked lissencephaly with ambiguous genitalia). However, patients showing one or more of these particular features were not incorporated in the present survey. On the contrary, mutations that result in a poly A expansion account for 59% of *ARX* mutations encountered in familial or isolated *ARX*-related cases [10]. Considering that our multiplex-PCR method is able to pinpoint deletions as well as expansions in the first two poly A stretches and that the latter are known to be the most frequent cause of *ARX*-related disorders, we can assume that the vast majority of the *ARX*-related variants of the test group male samples had been assessed.

**Figure 5:**
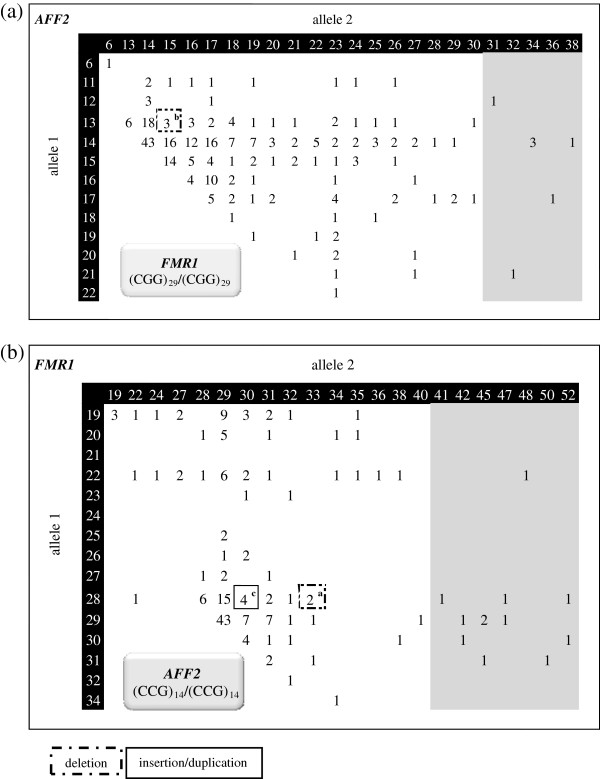
**Test group results.** Number of female homoallelic samples *per* allele profile of **(a)***FMR1* [(CGG)_29_/(CGG)_29_] and **(b)***AFF2* [(CCG)_14_/(CCG)_14_]. Intermediate alleles are grey shaded. Cases with different *ARX* variants are surrounded by boxes. *ARX* variant type: ^a^6 bp deletion; ^b^15 bp deletion; ^c^24 bp insertion/duplication.

In the females group, that represents 35.6% (n = 1780) of the analysed subjects, the allelic range of both *FMR1* and *AFF2* genes was similar to that obtained in male samples including several *FMR1* or *AFF2* intermediate-sized alleles. Southern blot for the analysis of *FMR1* or *AFF2* genes was carried out to discard a premutation or a full mutation in the case of an apparently homozygous women. Simultaneous *FMR1* and *AFF2* homoallelic samples were found in 5.8% of the female subjects (n = 103) whereas private homoallelism for *FMR1* or *AFF2* genes, occurred respectively in 27.5% (n = 490) and 17.8% (n = 317) of the samples [Figure 2(h)]. Considering the homoallelic females, the pairs [(CGG)_29_/(CGG)_29_] and [(CCG)_14_/(CCG)_14_] were observed in 73.4% and 71.8% of samples, respectively. As such, the results obtained in these two subsets of samples were used for an overall representation of the female samples [Figure 5(a) and (b) respectively]. In the females group, the number of *ARX* variants, was, as expected, significantly less than that observed in males: four size-variants of which three are deletions, ranging from 6 to 15 bp, and one is an insertion/duplication of 24 bp. The sample with a 15 bp deletion in *ARX* exon 2 has the following allelic profile [(CGG)_29_/(CGG)_30_] and [(CCG)_15_/(CCG)_17_, while the remaining *ARX* variants occurred in *FMR1* or *AFF2* homoallelic samples [Figure 4(d) and Figure 5(a) and (b)]. The pathogenic status of these variants, all occurring in a heterozygous state, remains to be determined.

Our test group includes subjects referred for FXS confirmation/exclusion, in the early 90s, classified as having a normal *FMR1* profile (by Southern blot). However, two *FMR1* premutations were now identified in a male (58 CGGs) and a female case [(CGG)_47_/(CGG)_61_] [Figure 2(e), Figure 3 and Figure 4(e) and (f)]. The blot was repeated using non-isotopic labelling, where these fragments were evident. We thus attributed the initial discrepancy to the relatively small size of the premutations, difficult to resolve under the older electrophoretic and labelling conditions. Despite Southern blotting and hybridization being the gold standard for molecular diagnosis of FXS and detection of mosaics, our results clearly illustrate the contribution of a PCR screening, particularly since it facilitates the detection and size-quantification of intermediate and premutated alleles.

The transition from an intermediate-sized allele to a full mutation in a single transmission was never observed, although changes in size can occur, particularly in female meiosis, which adds instability to those alleles [23]. The identification of these, probably unstable, alleles is of extreme importance since appropriate genetic counselling can be offered. Therefore, the benefits of the screening test are not limited to the subject tested but also are extended to other family members. Another added value concerns the *FMR1* premutation carriers that, until recently, were not considered at risk for any clinical disorder, however nowadays the role of these alleles in terms of female reproduction, namely in fragile X-associated primary ovarian insufficiency (FXPOI) cases and, in older individuals, in fragile X–associated tremor/ataxia syndrome (FXTAS) has been highlighted [24,25].

Based on our experience in FXS testing, one can estimate that around 3.5% of male and ~1% of female samples will not amplify the *FMR1* CGG repetitive region due to a mutation. Although unsuccessful amplification can be attributed to several factors (e.g. SNP in primer ligation site, deletion or technical pitfall) we do not have any such examples in our records as all these cases had a confirmed full mutation or large-premutation or both, confirmed by Southern blotting. Absence of amplification can occur due to the same factors described above in *AFF2* or *ARX* testing. For these two genes no estimates can be made as a larger cohort, specifically referred due to intellectual disability, has to be analysed.

## Conclusion

Overall, this multiplex-PCR assay can be beneficially used as a screening assay for *FMR1* gene premutations under 110 CGGs, *AFF2* gene premutations under 70 CCGs and *ARX* exon 2 size-variants. This methodology allows the screening of a considerable number of male and female samples with the same sensitivity/specificity as the simplex methodology and an enhanced accuracy when compared to Southern blot. Based on our results, 0.031% of male size-mosaics might be unnoticed. In addition, this methodology will not detect very rare cases of point mutations or duplications/deletions outside the hotspots of the three genes. In females, this screening shall unravel ~70% of *FMR1* and ~80% of *AFF2* gene analysis. Final mandatory diagnosis of homoallelic cases, size and methylation mosaics, X-chromosome aneuploidies, or *FMR1*, *AFF2* and *ARX* gene mutation are not aims of this methodology and must, thus, be achieved with other appropriate diagnostic techniques (e.g. Southern blotting, sequencing, methylation-specific PCR/MLPA or karyotyping). To overcome the putative overlap between the *ARX* fragment and an *AFF2* allele, an improvement would be the use of NED™ labelling instead of HEX, in either the *ARX* or the *AFF2* primer. Besides being rapid and cost-effective, this multiplex-PCR assay proved to be suitable as a high throughput screening strategy covering the previously mentioned XLID related conditions, representing a step forward in guiding the molecular approach and ultimately patient management, particularly in cases with difficult clinical ascertainment. This is definitely the case of *ARX* gene mutations that include different mutational phenomena but also a clear hotspot that accounts for at least 60% of *ARX* mutations [26,27]. In reports of patients with no obvious phenotype this number is usually increased. In fact, we found seventeen *ARX* variants in our cohort of patients that showed no obvious phenotypic feature. In conclusion, laboratories performing molecular genetic studies of idiopathic intellectual disability would benefit from the implementation of this methodology, which enables a widened scope of detection of the mutational hotspot regions of *FMR1*, *AFF2* and *ARX* genes in a single initial assay.

## Abbreviations

ARX ex2p: Portion of the second exon of *ARX* gene; bp: Base pairs; FRAXE: Fragile site “E”, implicated in intellectual disability; FXPOI: Fragile X-associated primary ovarian insufficiency; FXS: Fragile X syndrome; FXTAS: Fragile X–associated tremor/ataxia syndrome; gDNA: Genomic DNA; ID: Intellectual disability; MLPA: Multiplex ligation-dependent probe amplification; SNP: Single-nucleotide polymorphism; XLID: X-linked intellectual disability.

## Competing interests

The authors declare that they have no competing interests.

## Authors’ contributions

PJ was responsible for the study design, data analysis and manuscript preparation; BO genotyped the vast majority of samples and contributed to the write-up of the manuscript; IM established and standardized the methodology, collaborated in data analysis and presentation of results; RS responsible for the overall study and critical review of the manuscript. All authors have read and approved the final manuscript.

## Pre-publication history

The pre-publication history for this paper can be accessed here:

http://www.biomedcentral.com/1471-2350/14/80/prepub
